# Area-Level Socioeconomic Gradients in Overweight and Obesity in a Community-Derived Cohort of Health Service Users – A Cross-Sectional Study

**DOI:** 10.1371/journal.pone.0137261

**Published:** 2015-08-28

**Authors:** Andrew Bonney, Darren J. Mayne, Bryan D. Jones, Lawrence Bott, Stephen E. J. Andersen, Peter Caputi, Kathryn M. Weston, Don C. Iverson

**Affiliations:** 1 University of Wollongong, Graduate School of Medicine, Wollongong, New South Wales, 2522, Australia; 2 Public Health, Illawarra Shoalhaven Local Health District, Wollongong, New South Wales, 2500, Australia; 3 Illawarra Health and Medical Research Institute, University of Wollongong, New South Wales, 2522, Australia; 4 Sydney School of Public Health, The University of Sydney, Sydney, New South Wales, 2006, Australia; 5 Sonic Healthcare Ltd, North Ryde, New South Wales, 2113, Australia; 6 Southern.IML Pathology, Wollongong, New South Wales, 2500, Australia; 7 University of Wollongong, Centre for Health Initiatives, Wollongong, New South Wales, 2522, Australia; 8 Swinburne University of Technology, Faculty of Health, Arts and Design, Hawthorne, Victoria, 3122, Australia; Old Dominion University, UNITED STATES

## Abstract

**Background:**

Overweight and obesity lead to higher probability of individuals accessing primary care but adiposity estimates are rarely available at regional levels to inform health service planning. This paper analyses a large, community-derived clinical database of objectively measured body mass index (BMI) to explore relationships with area-level socioeconomic disadvantage for informing regional level planning activities.

**Materials and Methods:**

The study included 91776 adults who had BMI objectively measured between 1 July 2009 and 30 June 2011 by a single pathology provider. Demographic data and BMI were extracted and matched to 2006 national census socioeconomic data using geocoding. Adjusted odds-ratios for overweight and obesity were calculated using sex-stratified logistic regression models with socioeconomic disadvantage of census collection district of residence as the independent variable.

**Results:**

The prevalence of overweight or obesity was 79.2% (males) and 65.8% (females); increased with age to 74 years; and was higher in rural (74%) versus urban areas (71.4%) (p<0.001). Increasing socioeconomic disadvantage was associated with increasing prevalence of overweight (p<0.0001), obesity (p<0.0001) and overweight or obesity (p<0.0001) in women and obesity (p<0.0001) in men. Socioeconomic disadvantage was unrelated to overweight (p = 0.2024) and overweight or obesity (p = 0.4896) in males.

**Conclusion:**

It is feasible to link routinely-collected clinical data, representative of a discrete population, with geographic distribution of disadvantage, and to obtain meaningful area-level information useful for targeting interventions to improve population health. Our results demonstrate novel area-level socioeconomic gradients in overweight and obesity relevant to regional health service planning.

## Introduction

Australia has a major public health problem with overweight and obesity.[1] Using measured data, 63.4% of Australians were classified as overweight or obese in 2011–12.[2] Moreover, demonstrating entrenched community patterns, the proportion of Australians overweight or obese has increased from 56% in 1995.[2] An indication of the impact of these trends on the population’s health was evidenced in a recent report that high body mass index (BMI) had overtaken tobacco as the leading independent contributor to the burden of disease in areas of Australia.[3]

With appreciation of the complex interaction of factors involved, there has been growing international interest in the social and geographic influences in the development of obesity.[4,5] Broadly reflecting the international literature,[6–8] the evidence from Australian research has indicated that, while individual-level socioeconomic assets such as education and income are associated with lower BMI,[9] and independently of area-level disadvantage in women,[10,11] indicators of area-level, or geographic, socioeconomic disadvantage have been shown to be significant correlates of high BMI in Australian adults.[12,13].

While the association between area-level socioeconomic disadvantage and BMI should be of interest to public health policy makers and practitioners,[12] it is also significant for health service planning. Overweight and obesity, with their attendant chronic metabolic, cardiovascular and cancer-related health complications,[14,15] lead to higher probability of individuals accessing primary care services.[16] These primary care services are inextricably linked by geography to the individuals and communities they serve.[17] Thus, awareness of local area socioeconomic disadvantage and associated obesity risk are critical to building local capacity in chronic disease management and prevention, and to influencing strategic approaches to provision of requisite community health services. However, in Australia, area-level planning for overweight and obesity is inhibited by a lack of information about its epidemiology within specific communities or regions. Population obesity rates in New South Wales (NSW), Australia, are monitored by the Continuous Health Survey which provides reliable estimates for NSW and its local health districts, but not for smaller geographic areas,[18] a situation that could be problematic as overweight and obesity patterns in small areas may differ from those in larger areas.[19]

A comprehensive approach to preventing overweight and obesity, and area-level management of its health risks, presents an urgent and complex task.[20] Clearly, integrating both public health and primary care efforts is required, an approach to improving population health which has recently been advocated.[21] The use of geographically-linked, routinely-collected area-level data from the region of interest may be very useful for this purpose.[22,23] These data would have the advantages of being contemporaneous, cost-efficient and having sufficient population coverage to provide useful spatial resolution. However, despite the potential value of geographically-enabled clinical data for local health planning, its effective use by local health planners and clinical teams is hampered by lack of time, skills, financial resources and access to appropriate analytic mapping tools.[17]

Responding to these concerns, this paper describes the use of a large, longitudinal, community-derived clinical database to explore relationships between objectively-measured BMI and area-level socioeconomic disadvantage.

The specific objectives of this research were to:

1. Establish the feasibility of using a pre-existing clinical database for geographically-enabled analysis of BMI, and
2. Demonstrate the effectiveness of using a large community-derived database to investigate associations between BMI and area-level socioeconomic disadvantage in a sample of adult health service users in a discrete area of regional Australia, for use for health planning purposes.

## Materials and Methods

### Data sources and acquisition

This study was approved by the University of Wollongong and Illawarra Shoalhaven Local Health District (ISLHD) Health and Medical Human Research Ethics Committee (HE11/251).

**Fig 1:**
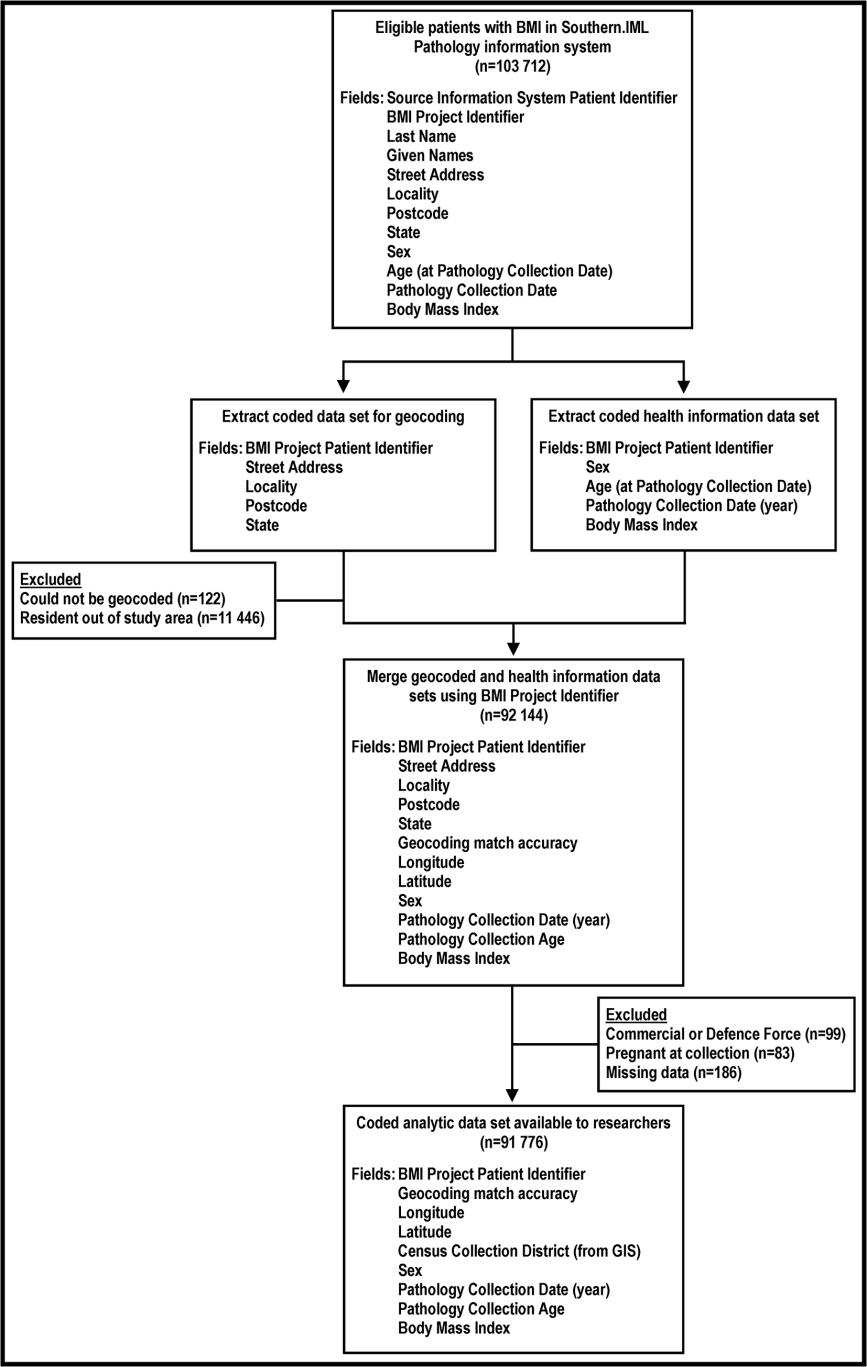
Data acquisition flow diagram.

Written informed consent was not given by participants for their clinical records to be used in this study; however, patient information was anonymized and de-identified prior to analysis, as follows. Data extraction and management were undertaken on-site at Southern.IML Pathology by one of the researchers who is also a senior staff member of Southern.IML (BDJ). Demographic (age, sex), anthropomorphic (BMI), pathology servicing (testing date) and location (residential address at testing) data were obtained from Southern.IML Pathology’s clinical management and reporting database. A unique, project-specific identifier was assigned to the pathology records of each patient and two coded datasets were extracted. The first contained only the project identifier and residential address (street, locality, postal code, state) for each patient. Using Quicklocate 3(G-NAF) software (MapData Services P/L, Greenwich, Australia), addresses were geocoded to assign longitude and latitude coordinates to identify individuals within the study area and assign small-area identifiers for matching to contextual variables from the 2006 Australian Census of Population and Housing.[24] Project identifiers for patients geocoded to the study area were then used to extract a second, coded analytic dataset containing only the project identifier, age, sex, year of testing, BMI, and longitude and latitude variables. As this dataset remained potentially re-identifiable through the latitude and longitude co-ordinates, it was stored on a secure network resource at Southern.IML Pathology, only accessed on-site, and analysed by researchers under the supervision of pathology company personnel. Exclusion criteria were pregnancy, resident outside the Illawarra-Shoalhaven local health district, having an address geocoded to commercial or Defence Force premises, or missing data on study variables. Out-of-area participants were excluded because the research ethics approval was limited to ISLHD residents aged ≥18 years only. Fig 1 outlines the data acquisition process.

### Study area and sample

This study was undertaken in the Illawarra-Shoalhaven region of NSW, Australia, which comprises the Kiama, Shellharbour, Shoalhaven and Wollongong local government areas (LGAs), covers a land mass of 5615 square kilometres and had an estimated resident population of 389157 on 30 June 2010.[25] The study cohort included all residents of the study area aged 18 years and over who attended a Southern.IML Pathology collection centre between 1 July 2009 and 30 June 2011 and had their BMI objectively measured. Southern.IML Pathology is the largest provider of private pathology services in the study area and routinely collects BMI on patients being tested for glomerular filtration rate and liver function; cholesterol (lipids), triglycerides, high and low density lipoproteins; glucose and glucose tolerance; urea electrolytes and creatinine; and 24-hour urine analyses.

### Study design and variables

A cross-sectional, hierarchical design was used to account for clustering within 2006 Australian Census of Population and Housing Census Collection Districts (CCDs). CCDs were the smallest geographical units at which statistical outputs were produced for the 2006 census and were used to assign area-level socioeconomic and geographic variables to cohort members.[26] In 2006, the study area comprised 631 inhabited and conterminous CCDs with a median land area of 0.4 square kilometres, 196 dwellings and 524 residents. The median number of cohort members per CCD was 136 (inter-quartile range = 85–187). One CCD contained no cohort members, and a second CCD contained no female cohort members.

### Outcome variables

The primary outcome measure was objectively-measured BMI categorised according to World Health Organisation BMI classification[27]: underweight (<18.5kg/m^2^); normal weight (18.5 to <25kg/m^2^); and overweight (25 to <30kg/m^2^) or obese (≥30kg/m^2^). Height and weight were collected at time of presentation using a standard procedure. Weight was measured clothed with emptied pockets on medical scales calibrated to measure within 500grams. Height was measured barefoot using stadiometers. BMI was calculated in the pathology information system as per routine practice using the formula mass(kg)/height(metres)^2^. Calculated BMIs for patients were included in the final analytic dataset, but not weights or heights. Analyses were limited to the most recent pathology episode for each patient in the study period to ensure their geocoded address and presentation address were spatially aligned.

### Study variable

The study variable was the 2006 CCD Index of Relative Socioeconomic Disadvantage (IRSD) quintile for a patient’s residence at time of pathology visit. The IRSD is an aggregated scale with components of income, education, employment, family structure, disability, housing, transport and internet connection.[28] IRSD is scaled across Australia to have a mean of 1000 and standard deviation of 1000. Higher IRSD scores for CCDs indicate relative lower socioeconomic disadvantage compared to CCDs with lower IRSD scores.

### Co-variates

Analyses were adjusted for gender, age (18–24, 25–34, 35–44, 45–54, 55–64, 65–74, ≥75 years) and geographic area of residence. Geographic area of residence was assigned using 2006 Australian Standard Geographic Classification Remoteness Areas (RA), which classify localities based on road distance to five service centre types.[29] We categorised the Major Metropolitan (RA0) LGAs of Kiama, Shellharbour and Wollongong as “urban” and the Inner Regional (RA1) LGA of Shoalhaven as “rural”.

### Statistical analysis

Patients were assigned the IRSD quintile value [28] from the 2006 Australian Census of Population and Housing for the CCD [30] in which their geocoded address was located at the time of pathology collection. Prevalence rates for BMI categories were calculated by gender, age group, IRSD quintile and RA of residence (i.e. urban or rural).[29] Adjusted odds for being overweight (excluding obese), obese (excluding overweight), and overweight or obese were calculated relative to being under or normal weight using generalised estimating equations at the CCD level with logit link functions, exchangeable correlation structures and IRSD quintile as the independent variable. Within-cohort relative risks expressed as odds-ratios provide robust indications of the direction of exposure-outcome relationships which are generalisable to the target population, even in non-representative samples.[30,31] We tested for effect modification of the study variable by covariates for all study outcomes and refit stratified models in the presence of interaction to aid interpretation. Statistical significance was assessed using the likelihood ratio test and an alpha level of 0.05. Data management and analysis were performed using The SAS System 9.3s (SAS Institute Inc. Cary, NC, USA) and Esri ArcGIS for Desktop version 10 (Esri, Redlands, CA, USA).

## Results

Body mass index (BMI) data were available for 103712 unique individuals aged 18 years and over. One hundred and twenty-two (0.1%) could not be geocoded (geocoding rate:99.9%) and 11446 (11.0%) were geocoded to an address outside the study area. Of the remaining 92144 Illawarra-Shoalhaven residents, 99 (0.1%) were geocoded to commercial or Defence Force facilities, 83 (0.1%) were pregnant, and 186 (0.2%) had missing study data, leaving a final analytic dataset after their exclusion of 91776 unique individuals, of which 5.9%, 16.7%, 24.8% and 52.6% were geocoded to the Kiama, Shellharbour, Shoalhaven and Wollongong LGAs respectively. Table 1 shows geocoding accuracy rates by LGA for the residents included in the final analytic dataset.

**Table 1:** Geocoding accuracy rates for included Illawarra-Shoalhaven residents by local government area (LGA).

Accuracy level	Kiama	Shellharbour	Shoalhaven	Wollongong	Total
	n	n	n	n	n
	(% accuracy)	(% accuracy)	(% accuracy)	(% accuracy)	(% accuracy)
**Address**	5066	14406	20713	45982	86167
	(93.8%)	(93.8%)	(91.2%)	(95.2%)	(93.9%)
**Street**	121	368	518	648	1655
	(2.2%)	(2.4%)	(2.3%)	(1.3%)	(1.8%)
**Locality**	215	583	1 489	1667	3954
	(4.0%)	(3.8%)	(6.6%)	(3.5%)	(4.3%)
**Total**	5402	15357	22720	48297	91776

The mean age of the cohort was 55.2 (SD = 15.66) years; 53.3% were female, and 24.8% lived in a RA1 area. Proportionally more cohort members (45%) were from less socioeconomically disadvantaged areas: quintile 1 (most disadvantaged):18.0%, quintile 2:18.5%, quintile 3 (middling disadvantage):18.5%, quintile 4:20.4%, quintile 5 (least disadvantaged):24.6%.

### Body Mass Index (BMI)

The mean BMI was 28.3kg/m^2^ for males (SD = 6.4) and 28.7kg/m^2^ for females (SD = 4.90). The distribution of body mass was significantly different for males compared with females (p<0.001), across age groups (p<0.001), IRSD quintiles (p<0.001) and RA location (p<0.001). Overall, the prevalence of being overweight or obese was 79.2% (males) and 65.8% (females); increased with increasing age to 74 years; increased with increasing socioeconomic disadvantage; and was marginally higher in rural (74%) than urban areas (71.4%). The cohort prevalence of underweight, normal weight, overweight and obesity by sex, ten-year age groups, area-level socioeconomic disadvantage and residential area are shown in Table 2.

**Table 2:** Characteristics of sample (n = 91776) by body mass index (BMI) class.

	Total	Underweight		Normal weight		Overweight		Obese		Overweight or obese	
		n	%	n	%	n	%	n	%	n	%
**Sex** *											
Males	42844	229	0.5	8695	20.3	19092	44.6	14828	34.6	33920	79.2
Female	48932	868	1.8	15846	32.4	15484	31.6	16734	34.2	32218	65.8
**Age group** *											
18–24	3796	219	5.8	2029	53.5	892	23.5	656	17.3	1548	40.8
25–34	6942	175	2.5	2709	39.0	2204	31.7	1854	26.7	4058	58.5
35–44	11693	126	1.1	3686	31.5	4089	35.0	3792	32.4	7881	67.4
45–54	19097	163	0.9	5053	26.5	7121	37.3	6760	35.4	13881	72.7
55–64	21917	141	0.6	4682	21.4	8571	39.1	8523	38.9	17094	78.0
65–74	18608	145	0.8	3734	20.1	7626	41.0	7103	38.2	14729	79.2
≥75	9723	128	1.3	2648	27.2	4073	41.9	2874	29.6	6947	71.4
**IRSD** ^a^ *											
1 high	1 547	217	1.3	3681	22.2	5901	35.7	6748	40.8	12649	76.4
2	16993	233	1.4	4337	25.5	6159	36.2	6264	36.9	12423	73.1
3 middling	16982	204	1.2	4590	27.0	6435	37.9	5753	33.9	12188	71.8
4	18672	202	1.1	5215	27.9	7265	38.9	5990	32.1	13255	71.0
5 low	22582	241	1.1	6718	29.7	8816	39.0	6807	30.1	15623	69.2
**Residential area** *											
Rural	22720	264	1.2	5645	24.8	8721	38.4	8090	35.6	16811	74.0
Urban	69056	833	1.2	18896	27.4	25855	37.4	23472	34.0	49327	71.4

* p<0.001

IRSD^a^, Index of Relative Socioeconomic Disadvantage

### Socioeconomic disadvantage and BMI

Table 3 shows unadjusted and adjusted estimates for overweight, obese, and overweight or obese models. In unadjusted analyses, increasing socioeconomic disadvantage of residential CCD was associated with increased odds of being overweight (p = 0.0002), obese (p<0.0001), and overweight or obese (p<0.0001). However, there was very strong evidence of effect modification of BMI-IRSD associations in multivariable adjustment models (p<0.0001 for all interactions) consistent with stronger gradients for females compared to males (see Table 3).

**Table 3:** Adjusted and unadjusted odds-ratios for overweight, obese, and overweight or obese body mass categories.

	Overweight (25.0–29.9 kg/m^2^)		Obese (≥30.0 kg/m^2^)		Overweight or obese (≥25.0 kg/m^2^)	
	Unadjusted OR^a^	Adjusted OR	Unadjusted OR	Adjusted OR	Unadjusted OR	Adjusted OR
IRSD^b^ Quintile	p = 0.0002	p = 0.0002	p<0.0001	p<0.0001	p<0.0001	p<0.0001
1 –high	1.19 (1.10–1.27)	0.79 (0.68–0.91)	1.75 (1.59–1.91)	1.31 (1.12–1.54)	1.43 (1.33–1.54)	1.01 (0.88–1.15)
2	1.07 (1.00–1.15)	0.82 (0.72–0.93)	1.42 (1.29–1.56)	1.21 (1.04–1.40)	1.22 (1.13–1.32)	0.98 (0.88–1.15)
3—middling	1.06 (0.99–1.14)	0.87 (0.76–1.00)	1.23 (1.11–1.35)	1.10 (0.93–1.30)	1.13 (1.05–1.22)	0.97 (0.85–1.11)
4	1.07 (1.00–1.14)	0.94 (0.82–1.06)	1.13 (1.02–1.25)	1.05 (0.90–1.23)	1.10 (1.02–1.19)	0.99 (0.87–1.12)
5 –low	1.00	1.00	1.00	1.00	1.00	1.00
Sex	p<0.0001	p<0.0001	p<0.0001	p<0.0001	p<0.0001	p<0.0001
Male	1.00	1.00	1.00	1.00	1.00	1.00
Female	0.43 (0.42–0.45)	0.37 (0.34–0.40)	0.61 (0.59–0.63)	0.53 (0.49–0.57)	0.51 (0.49–0.53)	0.43 (0.40–0.46)
Age group	p<0.0001	p<0.0001	p<0.0001	p<0.0001	p<0.0001	p<0.0001
18–24	0.29 (0.27–0.32)	0.25 (0.21–0.29)	0.23 (0.21–0.26)	0.23 (0.19–0.28)	0.26 (0.24–0.28)	0.25 (0.22–0.28)
25–34	0.56 (0.52–0.60)	0.52 (0.46–0.59)	0.49 (0.46–0.53)	0.48 (0.41–0.55)	0.52 (0.49–0.55)	0.51 (0.45–0.56)
35–44	0.78 (0.74–0.83)	0.77 (0.70–0.85)	0.76 (0.72–0.81)	0.72 (0.65–0.80)	0.77 (0.74–0.81)	0.75 (0.69–0.82)
45–54	1.00	1.00	1.00	1.00	1.00	1.00
55–64	1.31 (1.24–1.37)	1.18 (1.07–1.31)	1.35 (1.29–0.81)	1.26 (1.14–1.39)	1.33 (1.27–1.39)	1.22 (1.12–1.33)
65–74	1.44 (1.36–1.52)	1.21 (1.09–1.35)	1.37 (1.30–1.45)	1.30 (1.17–1.45)	1.41 (1.34–1.48)	1.28 (1.16–1.41)
75+	1.08 (1.01–1.14)	0.90 (0.78–1.03)	0.79 (0.74–0.85)	0.79 (0.66–0.94)	0.94 (0.88–0.99)	0.88 (0.77–1.00)
Residential area	p<0.0001	p = 0.6766	p = 0.0011	p = 0.2572	p<0.0001	p = 0.6819
Metropolitan	1.00	1.00	1.00	1.00	1.00	1.00
Rural	1.13 (1.07–1.18)	1.11 (0.97–1.27)	1.13 (1.05–1.21)	1.17 (0.96–1.420	1.13 (1.07–1.20)	1.17 (1.00–1.38)
Effect modification						
IRSD *Sex		p<0.0001		p<0.0001		p<0.0001
1 –high (female)		1.57 (1.41–1.75)		1.59 (1.42–1.78)		1.63 (1.48–1.80)
2 (female)		1.35 (1.21–1.50)		1.24 (1.11–1.38)		1.31 (1.19–1.45)
3 –middling (female)		1.22 (1.09–1.36)		1.19 (1.06–1.33)		1.21 (1.09–1.35)
4 (female)		1.17 (1.05–1.30)		1.12 (0.99–1.27)		1.15 (1.04–1.27)
5 –low (female)		1.00		1.00		1.00
IRSD *Age group		p = 0.1691		p = 0.3818		p = 0.5027
IRSD *Residential area		p = 0.3181		p = 0.1870		p = 0.2055
ICC^c^	0.008	0.008	0.025	0.024	0.012	0.012

Adjusted odds ratios for relative socioeconomic disadvantage quintiles calculated using generalised estimating equations.

Intraclass correlation coefficients reported for unadjusted parameter estimates are for relative socioeconomic disadvantage (IRSD) models.

Obese individuals were excluded from overweight analyses and overweight individuals were excluded from obese analyses.

OR^a^, Odds Ratio

IRSD^b^, Index of Relative Socioeconomic Disadvantage

ICC^c^, Intra-cluster Correlation Coefficient

Table 4 reports sex-stratified odds-ratios of being overweight, obese, and overweight or obese for socioeconomic disadvantage of residential CCD adjusted for age and RA at pathology collection. Increasing socioeconomic disadvantage of residential CCD was associated with increasing prevalence of obesity in men (p<0.0001) but was unrelated to either overweight (p = 0.2024) or overweight or obese (p = 0.4896) body mass categories. The odds of obesity for men in the most socioeconomically disadvantaged quintile were increased by a factor of 1.37 (95% CI 1.22–1.55) compared to men in the least socioeconomically disadvantaged quintile. In contrast, increasing socioeconomic disadvantage of residential CCD for women was associated with increased prevalence of all study outcomes: overweight (p<0.0001), obesity (p<0.0001), and overweight or obesity (p<0.0001). Compared to women resident in the least disadvantaged CCDs, women living in the most disadvantaged CCDs had an odds-ratio for overweight of 1.37 (1.25–1.50), obesity of 2.06 (95% CI 1.84–2.30) and overweight or obesity of 1.71 (95% CI 1.56–1.87).

**Table 4:** Adjusted socioeconomic disadvantage odds-ratios for overweight, obese, and overweight or obese body mass categories by sex.

IRSD^a^ quintile	Overweight (25.0–29.9 kg/m^2^)				Obese (≥30.0 kg/m^2^)				Overweight or obese (≥25.0 kg/m^2^)			
	n^b^	Total	%	Adjusted OR^c^	N	Total	%	Adjusted OR	N	Total	%	Adjusted OR
Males				p = 0.2024				p<0.0001				p = 0.4896
1—high	3136	4657	67.3	0.93 (0.84–1.02)	2997	4518	66.3	1.37 (1.22–1.55)	6133	7654	80.1	1.11 (1.01–1.23)
2	3359	5020	66.9	0.90 (0.82–0.99)	2982	4643	64.2	1.28 (1.14–1.43)	6341	8002	79.2	1.05 (0.96–1.16)
3—middling	3530	5179	68.2	0.96 (0.88–1.06)	2676	4325	61.9	1.14 (1.01–1.28)	6206	7855	79.0	1.03 (0.93–1.14)
4	4030	5886	68.5	0.97 (0.89–1.06)	2869	4725	60.7	1.05 (0.93–1.19)	6899	8755	78.8	1.01 (0.92–1.11)
5 –low	5037	7274	69.2	Referent	3304	5541	59.6	Referent	8341	10578	78.9	Referent
ICC^d^				0.008				0.027				0.010
Females				p<0.0001				p<0.0001				p<0.0001
1—high	2765	5142	53.8	1.37 (1.25–1.50)	3751	6128	61.2	2.06 (1.84–2.30)	6516	8893	73.3	1.71 (1.56–1.87)
2	2800	5709	49.0	1.16 (10.6–1.27)	3282	6191	53.0	1.50 (1.35–1.68)	6082	8991	67.6	1.32 (1.20–1.45)
3—middling	2905	6050	48.0	1.15 (1.05–1.25)	3077	6222	49.5	1.33 (1.19–1.48)	5982	9127	65.5	1.23 (1.12–1.34)
4	3235	6796	47.6	1.11 (1.01–1.21)	3121	6682	46.7	1.17 (1.04–1.32)	6356	9917	64.1	1.14 (1.03–1.25)
5 –low	3779	8501	44.5	Referent	3503	8225	42.6	Referent	7282	12004	60.7	Referent
ICC				0.010				0.026				

IRSD^a^, Index of Relative Socioeconomic Disadvantage

n^b^, number with outcome in quintile; Total, total number in quintile; %, percent of quintile with outcome

OR^c^, Odds Ratio

ICC^d^, Intra-cluster Correlation Coefficient

Odds ratios for relative socioeconomic disadvantage quintiles calculated using sex-stratified generalised estimating equations adjusted for age and residential location at pathology collection.

Obese individuals were excluded from overweight analyses and overweight individuals were excluded from obese analyses.

Intra-cluster Correlation Coefficients (ICC) for multivariable models are reported in Tables 3 and 4 and averaged 0.9% for overweight, 2.6% for obesity and 1.2% for overweight and obesity, which were consistent with ICC estimates for weight-related physical measures in comparable primary health care settings.[32]

## Discussion

This study demonstrates it is feasible to link routinely-collected clinical data with geographic distribution of disadvantage. Tools to visualize IRSD in Australia across a variety of geographical statistical areas are publicly available and do not require specialised statistical skills.[33] Using such tools, meaningful information can be obtained to allow health service planners to anticipate and respond to demand for services geographically, as well as target preventive services and health promotion activities.[34]

We have described differing area-level socioeconomic gradients for overweight and obesity when these are considered as separate categories for men and women. The gradient was consistent for women across both categories of elevated BMI, but only present for obesity for men. We also observed a socioeconomic gradient when overweight and obese categories of BMI were combined for women but not for men due to the lack of a socioeconomic gradient for overweight in men. We are not aware of any previous reports of this observation using area-level socioeconomic indices in Australia. Using household income as the area-level socioeconomic indicator, King et al (2006) observed a graded increase in mean BMI from least to most disadvantaged areas for both sexes,[12] but did not analyse the data by BMI category. Analysing data from 16243 participants in the 2001 National Health Survey, Brown and Siahpush (2008) found that increasing area-level disadvantage measured by IRSD was associated with increased risk of overweight and obese BMI in females but not males.[13] It is possible our results reflect a change in the pattern of weight gain in the population in the decade since that study. Recent reports have indicated a slowing of weight gain across the population other than for older men and for those in areas of most socioeconomic disadvantage,[35] which may partly explain the socioeconomic gradients encountered in our findings. Brennan et al (2010) observed an increased risk of obesity in men resident in CCDs in the lower quintile of socioeconomic status as measured by the IRSD.[36] However, their analyses did not extend to comparisons of risk of overweight and obesity.[36] It is of interest to compare our findings with a recent Australian representative population study,[37] which demonstrated opposing socioeconomic gradients for overweight and obesity in men using total annual household income as an individual-level socioeconomic marker. Whereas these authors did not find a socioeconomic gradient for risk of overweight in women,[37] our data did indicate a gradient. The differences in our findings may be due to our larger sample size allowing detection of a gradient in women, or our choice of using an area-level socioeconomic indicator which included education along with income and other factors. Lower education attainment has been previously described as being associated with increased BMI in Australian men and women.[13] The inclusion of education as a factor may possibly explain the difference in our results from those using income alone; flattening the gradient produced by the association between higher income and overweight in men and producing a gradient in the association of area-level disadvantage and overweight in women. Systematic reviews have outlined a consistent pattern of inverse relationship between BMI and socioeconomic status in women in developed countries and less consistent socioeconomic gradients for men.[6–8] Our findings of differing gradients in overweight and obesity, along with results of others,[37] contribute to the observations of the inconsistent patterning observed for men and add to evidence that it is potentially misleading to aggregate overweight and obese BMI categories in socioeconomically-linked analyses.[37]

### Implications of the findings

The practical implication of these findings is their application for health planners with access to locally-derived data. Our data indicate, not unexpectedly, that for both men and women in the Illawarra-Shoalhaven region, the prevalence of obesity is higher in neighbourhoods of greatest disadvantage. However, for adult male health service users, the prevalence of overweight is similar in neighbourhoods of high and low disadvantage. Estimates of the relative risk or prevalence of overweight or obesity for health service users at a neighbourhood (i.e. CCD) level can inform targeted strategies towards prevention of illness, or management, individualised to localities, and addressing context-specific cultural and community factors.[1] For example, in neighbourhoods with high disadvantage, a combined approach of building capacity in obesity-related chronic disease management within community health services, integrated with nutrition education [38] and community-level activities to improve fresh food availability and the physical activity environment, may represent effective prioritisation of resources.[1,10] As men may be less likely to seek preventive health services,[39] a settings approach to weight management in workplaces[1] and increased awareness of the need to address BMI in opportunistic preventive health checks may more effectively target men and be more appropriate for health services in neighbourhoods with least disadvantage. Previous research has described how involving local health workers in interpreting geographically presented health data provided valuable insights into the data while engendering significant enthusiasm for a community-oriented health care approach.[17] Thus, there is significant scope for the use of geographically-enabled data to engage local primary health care services in the improvement of area-level population health. Further research including spatial clustering of BMI and associated health risks, relationships with proximity to community health services, and temporal changes, would significantly support population health efforts in discrete communities or regions.

### Limitations

These findings should be interpreted within the study limitations. The cross-sectional nature of the analyses does not facilitate attribution of causal relationships. The use of area-level indices alone did not permit control for individual-level attributes. Our study sample was derived from persons already using the health system and being referred for pathology testing, and is likely biased with a higher proportion of persons in ill-health and hence, a higher prevalence of health risk factors. This assumption appears validated by the higher prevalence of overweight or obesity in our sample compared with population estimates. The Australian Health Survey reported the prevalence of measured overweight or obesity as 70.3% (men) and 56.2% (women); compared with 79.2% and 65.8% respectively in our sample.[2] Hence, it is not appropriate to use our sample for estimating population point prevalence estimates.

## Conclusions

Routinely collected clinical data can inform community and regional health planning to combat overweight and obesity and their health complications. Knowledge of differential trends in overweight and obesity in association with area-level socioeconomic disadvantage will help to tailor and target interventions to assist in optimising the impact of health expenditure in improving population health.
